# A multi-level approach for the use of routinely collected patient-reported outcome measures (PROMs) data in healthcare systems

**DOI:** 10.1186/s41687-021-00375-1

**Published:** 2021-10-12

**Authors:** Fatima Al Sayah, Markus Lahtinen, Gouke J. Bonsel, Arto Ohinmaa, Jeffrey A. Johnson

**Affiliations:** 1 Alberta PROMs and EQ-5D Research and Support Unit (APERSU), School of Public Health, University of Alberta, 2-040 Li Ka Shing Centre for Health Research Innovation, Edmonton, Alberta, T6G 2E1 Canada; 2 Health Quality Council of Alberta, Calgary, Alberta, Canada; 3 EuroQol Research Foundation, Rotterdam, The Netherlands

## Introduction

Patient-reported outcome measures (PROMs) are instruments that measure health, including symptoms, functional status, health perceptions and health-related quality of life, as reported by patients themselves [1]. Today they gain importance in informing patients, caregivers, managers, and the public on the degree to which the goals of health care are attained. The introduction of PROMs into healthcare settings is not intended to replace existing outcome measures, but rather enhances health outcome measurement by making it more comprehensive and patient-centered. As such, PROMs data should be viewed as complimentary to various types of data traditionally collected in healthcare systems. The use of PROMs alongside clinician-reported outcomes and administrative data can better inform clinical and policy decisions. This underscores the importance of integrating PROMs into existing outcomes measurement systems and linking PROMs data with other patient data sources.

## Background

PROMs were originally developed for use in clinical effectiveness research as a means to incorporate patient’s voice in the assessment of clinical interventions [2]. Gradually, their use became more apparent in population health surveillance, then in clinical practice, and eventually into the healthcare system more broadly. While their use in clinical research is well established, many challenges persist with their use in other applications, particularly for routine outcome measurement within healthcare systems, which has increased in recent years. International initiatives, such as the International Consortium for Health Outcomes Measurement (ICHOM) and the Patient-reported Indicator Surveys (PaRIS) by the OECD, have accelerated the movement towards routine PROMs measurement within health systems. As a public activity, it has been led by the National Health Service (NHS) in England, employing routine collection of PROMs in four elective surgeries funded under an NHS PROMs program [3]. In this program, the EQ-5D was used as a generic PROM in all four surgeries, alongside disease-specific measures within each clinical area. In Sweden, PROMs measurement started in orthopaedics registries, and currently 90% of national quality registers routinely collect PROMs data, with the EQ-5D being the most commonly used measure [4]. In the US, PROMs are part of the Health Outcomes Survey, used by the Centres for Medicare and Medicaid Services to evaluate the Medicare Advantage programme [5]. In Canada, there are several national initiatives, led by the Canadian Institute of Health Information, with a focus on areas such as hip and knee arthroplasty, renal care and mental health [6]. There are also several provincial-level initiatives where PROMs are used in routine outcome measurement. For example, in Alberta, the EQ-5D is the recommended generic PROM for use in routine outcome measurement within the healthcare system, and has been incorporated into the electronic medical record, so that each patient accessing the system would be able to complete a PROM.

There is a number of published papers providing a framework and guidance for the collection and implementation of PROMs in clinical practice [7–9]. While these papers and others are very useful guides for users, they focus more on a particular application of PROMs and their implementation within the system, and less on the use of PROMs data obtained from routine measurement. With large-scale collection of PROMs data, it is essential to focus not only on approaches to enhance data collection and implementation within clinical workflow, but also on how to enhance the use of this routinely collected data within the healthcare system. The use of PROMs data within healthcare systems has primarily focused on either improving the clinical management of individual patients, as in the case of Sweden and the USA, or comparison of healthcare providers (organizations and individuals) in England [10].

Given the extensive efforts and resources that are required for a successful routine collection of PROMs within healthcare systems, we believe that the use of PROMs data should be maximized and employed for various purposes. Our aims were to provide an overview of PROMs implementation considerations based on the literature and real-world implementation, and propose a multi-level approach to enhancing the use of routinely collected PROMs data within healthcare systems. Our target audience includes clinicians, managers, healthcare system administrators, and policy makers who are involved in PROMs programs. This approach encourages users to take into account all potential usages of PROMs at various levels within the system during the planning and implementation phases of these programs.

## A brief overview on PROMs implementation

Research into the planning and implementation of PROMs within clinical settings has escalated over the last two decades. Currently, we have evidence that provides adequate guidance on ways to ensure successful implementation of PROMs [11–15], and on practical and resource-related factors that impact the use of PROMs within the healthcare system [11, 16, 17]. Context, however, remains a key determining factor of the successful implementation of PROMs in these settings [18]. In Alberta, and through our extensive work with and support for various PROMs programs’ leaders, we used implementation strategies identified in the literature and supplemented it with the local contextual experience of healthcare system users in the province. In Table 1, we provide a list of implementation considerations founded in evidence [7–9, 11, 19, 20] and real-world experience that have been shown to effectively support end-users in planning and implementing PROMs programs. We emphasize that this list of actions is not sequential, but rather is an iterative and interactive process that varies from one setting to another.

**Table 1:** PROMs Program Implementation Considerations

Setting up a implementation team	Establish a team that plans and implements the PROMs program is a key step towards success. It is advisable to have health care providers and patient representatives on the team, as well as PROMs experts to assist with technical aspects of the program. The committee composition will vary according to the setting
Identifying purpose	Identify a clear purpose for the PROMs program, which could include enhancing clinical practice (e.g., screening, monitoring), evaluating the impact of a healthcare program or service, evaluating the performance of healthcare providers, or a combination of one or more of these purposes. A clear purpose is crucial for identifying the end-users of PROMs data and for each of the subsequent steps
Identifying target population	Identify and describe the target group(s) of patients that will complete the measure(s) based on their clinical condition(s), age group(s), language, ability to self-report or proxy, and feasibility of various administration modes (in person, via phone, web-based). These factors should all be considered when selecting a PROM
Selecting a PROM(s)	The choice of PROM(s) is a delicate process whereby several factors have to be taken into account including the purpose of the measurement, measurement properties of available PROMs, patient acceptability, and a number of practical considerations (e.g., mode of administration, languages available, reference period, license fees). Multiple PROMs could be used in the same program; a combination between a generic and a disease-specific PROM is common
Using electronic tools	Where feasible and accessible, using electronic platforms for data collection minimizes clinical and administrative burden, ensures timely feedback of PROMs scores to clinicians and patients, and allows proper presentation of PROMs data to facilitate its interpretation. Ideally, integrating PROMs into electronic health records for the purposes of data collecting and reporting has been reported to be the most efficient and successful usage of these platforms
Change management	The introduction of PROMs into clinical settings presents a significant change to clinical workflows and to care practices that both providers and patients are accustomed to. Such changes vary from one setting to another depending on the presence of other similar initiatives. PROMs programs implementation teams need to assess the organizational readiness and identify factors and actions that would facilitate such changes to take place. This would influence the extent of training and education required, and the pace of program adoption
Education and training	This involves training all potential PROMs end-users within a given setting including providers, patients, and administrators, and includes explanation of the PROM(s) that will be used (e.g., construct being measured, response options), scores meaning and interpretation of their changes, and action items related to the PROM, as well as methods of collecting and using the data
Collecting PROMs data—pilot	Identify the timing, frequency and time window of measurements, as well as mode (e.g., paper-based, via phone, web-based) and place (e.g., home, clinic) of PROM(s) administration; these all depend on the purpose of measurement and the clinical setting where a PROM is implemented
Using PROMs data—pilot	Use PROMs data based on the purpose of the program (e.g., supporting clinical patient management, evaluating quality of healthcare services). The usefulness of data will depend on its quality, and on several previous identified factors (e.g., mode of data collection and reporting, user buy-in, missing data)
Reporting and feedback—pilot	Report PROMs data back to target users including providers, patients and administrators based on the intended purpose of the program. PROMs data reporting will vary by the intended user
Evaluation of pilot phase	A pilot phase has shown to be a very useful approach to evaluate various aspects of the PROMs program before widespread use in a given clinical setting. It allows evaluating education and training of the clinical teams, the mode, timing and frequency of data collection, the reporting and use of PROMs data by targeted users (e.g., clinicians, administrators), and other aspects of the program. Potential gaps or challenges identified in this phase could be addressed before widespread implementation
Upscale and spread PROMs program	After the pilot phase and implementation required revisions, the PROMs program could be scaled up to the entire target clinical setting and population. Incorporating PROMs into existing outcome measurement frameworks has been reported to ensure better uptake and sustainability of the program and better use of the data

The implementation considerations presented here are based on implementation guidelines and strategies reported in the literature [7–9, 11, 19, 20] and supplemented with our experience working with various health system users of PROMs in Alberta

## Use of routinely collected PROMs data: a multi-level approach

**Fig. 1:**
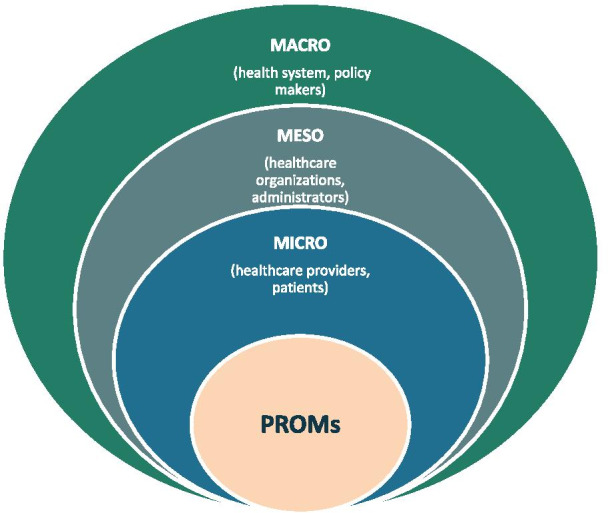
A multi-level approach for the use of PROMs data

PROMs data have the potential to inform stakeholders, enhance patient-centered care, enable individual decision-making, and contribute to evaluation and programming of services within real world health care delivery systems, as well as quality improvement initiatives [12, 15]. We propose a multi-level approach that would maximize the use of routinely collected PROMs data within healthcare systems (Fig. 1):

1. *Micro—Patient/clinician* At this level, PROMs data can inform clinical practice and enhance patient management. PROMs data could be used for screening, risk stratification and prognosis/expectation management, prioritization, goal setting, monitoring patients’ health status over time, and facilitating communication between patients and health care providers [15, 17, 18]. PROMs data could be used to incorporate patients’ voice in informing decision-making around their own care and clinical management by directly identifying health concerns assessed by the PROM, or via other tools such as patient decision aids that are developed using PROMs data.
2. *Meso—Organization level* At this level, PROMs data from a group of patients within an organization (e.g., clinic, hospital, treatment site) are aggregated and analyzed to assess and monitor patients’ health outcomes over time, evaluate health programs within certain clinics or organizations, or examine the effectiveness of healthcare services [7, 8]. PROMs data at this level can also be used to identify gaps in care and to triage patients based on self-reported health status or symptoms, which could be used to inform service delivery and programming.
3. *Macro—System level* At this level, PROMs data are aggregated to evaluate the performance of providers (individuals, organizations) at the system level by comparing outcomes across these providers as well as across different jurisdictions or regions covered by the healthcare system [10, 18]. This high-level use of PROMs data could support health policy-makers in healthcare allocation decisions that incorporate patients’ perspective and priorities, and emphasize patient-informed value-based care [21]. PROMs play a key role in current international reports on health care performance (like OECD report Health at a Glance 2019), where EQ-5D was acknowledged as a Rosetta Stone-like measure linking national registry outcome data on hip- and knee replacement [22].

Considering all these potential usages of PROMs data is crucial at the stage of planning the implementation of PROMs program. A summary of the use of PROMs data at each of these levels is presented in Table 2.

**Table 2:** Key usages of PROMs data at the micro, meso, and macro levels within the system

Micro	Meso	Macro
Patient and Clinician Informs patient management and care plan	Healthcare Organization Informs performance measurement and evaluation	Healthcare System Informs resource planning and allocation
Patient completes the PROM prior or during clinical encounter. The PROM could be used to: Identify health issues as reported by patients, especially those that may go unnoticed (e.g., pain, anxiety) Screen for specific symptoms or health problems Monitor patients' outcomes over time to inform treatment decisions Triage patients according to their self-reported health problems to inform care delivery Empower patients to share how they feel about their health with their care providers	PROM completed by multiple patients at various clinics are aggregated and data analyzed to: Assess and monitor patients' health outcomes at a group level Evaluate the performance of an organization in comparison with best practices and benchmarks Examine the effectiveness of a health intervention or program Establish benchmarks and best practices for providers (individuals) Measure outcomes as compared to cost and healthcare utilization at the organization level	PROM completed by multiple patients at various clinical sites are aggregated and data analyzed to: Compare providers’ performance across the healthcare system Compare patients' response to treatments or interventions across healthcare delivery sites Identify key health problems at the system level Establish benchmarks and best practices for providers (organizations) Measure outcomes as compared to cost and healthcare utilization at the system level

## Discussion

This multi-level approach presents a roadmap to enhance the use of PROMs data within the healthcare system. It intends to support end-users in understanding the potential usages of this data, and getting the most value out of routinely collected PROMs data.

Many interrelated factors impact the collection and use of PROMs data within healthcare systems. The success of using PROMs data at the micro level depends on many factors including clinicians’ training in using PROMs and their buy-in, the clinical usefulness of the PROM, the ease and timeliness of collecting and reporting PROMs data, integrating PROMs into clinical workflows and electronic health records, ease of interpreting PROMs data, and guidance to support clinical actions based on PROMs data [16, 23]. The use of aggregate PROMs data at the meso and macro levels is limited. Greenhalgh and colleagues suggests that there are three main theories driving the use of aggregated PROMs data in quality improvement initiatives, largely based on the NHS PROMs program, including supporting patient choice (selection pathway), improving accountability and enabling providers to compare their performance with others (change pathway) [18]. While less evidence is available to support the former pathway, emerging evidence supports the “change pathway” [18].

It is important to note that much of the work that takes place to implement PROMs programs in real world healthcare settings does not get published, partly due to restrictions related to patient privacy and consent for data use for research purposes. In a recent international meeting of healthcare system PROMs users, various successful examples of PROMs programs within healthcare systems were presented and discussed, however, most of these programs—with the exception of the NHS PROMs program—have limited or no publications reporting them [24]. In Alberta, the use of PROMs data at the micro, meso and macro levels vary across programs and clinical settings. For example, at Cancer Care Alberta, patients complete a PROM at each visit, then data are entered into a dashboard that a clinician can access during the clinical encounter and use to evaluate change in PROMs scores of a given patient over time [25]. This micro level use of PROMs data has been facilitated by extensive training of clinicians, and integrating PROMs collection and reporting into the clinical workflow of oncology clinics. Alternatively, at the Alberta Bone and Joint Health Institute, patients undergoing hip or knee arthroplasty complete a PROM before their surgery, and at 3 and 12 months post-surgery [25]. PROMs data are entered into a database, and analyzed at the aggregate level to evaluate the effectiveness of these surgical interventions, and to compare across providers and surgical sites, while clinicians do not have access to this data during clinical encounters limiting the micro level use of this data. Detailed information about the use of PROMs data within each of these levels in various clinical areas, and the facilitators and barriers to their use are provided in subsequent papers in this supplement.

The transition of PROMs use from clinical effectiveness research into real world healthcare setting, with all its variants, imposes various practical and methodological challenges on users [11, 12, 14]. For instance, in clinical studies, there are defined time points for data collection and a specified mode of administering PROMs that is often managed by research staff; however, the timing, frequency and completion of PROMs measurements may be challenging to maintain in real-world healthcare settings given various clinical workflows and other contextual factors that vary across settings. Further, in research applications, PROMs data are used at the aggregate level for the whole study sample with a very clear analytical purpose; however, in clinical settings, PROMs data are used both at the individual patient level and at the aggregate level. These practical challenges could be partly addressed with standardization of PROMs measurement and incorporating it into existing measurement frameworks and electronic medical records.

Using real-world routinely collected PROMs data also imposes several methodological challenges that users need to consider. These include attrition and missing data, varying time points of PROM(s) measurement, lack of a control arm in comparative effectiveness analysis, large data pitfalls, sample representativeness, statistical significance versus clinical importance, issues imposed by response shift in long-term data collection especially in chronic diseases, and case-mix adjustment, among others.

Despite significant developments in research and application of PROMs in real world settings in many countries around the world, more evidence and guidance for proper implementation and use of PROMs data is needed. Standards for the selection, collection, interpretation, and reporting of PROMs data with other clinical or administrative datasets are essential to ensure meaningful use of this data for clinical care and policy-decision-making. Future research should focus on approaches of integrating PROMs data with other patient data to enhance its use at all levels within the healthcare system.

## Data Availability

Not applicable.

## Abbreviations

PROM: Patient-Reported Outcome Measure
ICHOM: International Consortium for Health Outcomes Measurement
PaRIS: Patient-Reported Indicator Surveys
NHS: National Health Service
